# miRNA profiling of urinary exosomes to assess the progression of acute kidney injury

**DOI:** 10.1038/s41598-019-40747-8

**Published:** 2019-03-18

**Authors:** Hiroko Sonoda, Byung Rho Lee, Ki-Hoon Park, Deepak Nihalani, Je-Hyun Yoon, Masahiro Ikeda, Sang-Ho Kwon

**Affiliations:** 1 Department of Veterinary Pharmacology, University of Miyazaki, Miyazaki, 889-2192 Japan; 2 Department of Cellular Biology and Anatomy, Medical College of Georgia, Augusta University, Augusta, GA USA; 3 Department of Medicine, Nephrology Division, Medical University of South Carolina, Charleston, SC USA; 4 Department of Biochemistry and Molecular biology, Medical University of South Carolina, Charleston, SC USA

## Abstract

Because exosomes have gained attention as a source of biomarkers, we investigated if miRNAs in exosomes (exo-miRs) can report the disease progression of organ injury. Using rat renal ischemia-reperfusion injury (IRI) as a model of acute kidney injury (AKI), we determined temporally-released exo-miRs in urine during IRI and found that these exo-miRs could reliably mirror the progression of AKI. From the longitudinal measurements of miRNA expression in kidney and urine, we found that release of exo- miRs was a regulated sorting process. In the injury state, miR-16, miR-24, and miR-200c were increased in the urine. Interestingly, expression of target mRNAs of these exo-miRs was significantly altered in renal medulla. Next, in the early recovery state, exo-miRs (miR-9a, miR-141, miR-200a, miR-200c, miR-429), which share Zeb1/2 as a common target mRNA, were upregulated together, indicating that they reflect TGF-β-associated renal fibrosis. Finally, release of exo-miRs (miR-125a, miR-351) was regulated by TGF-β1 and was able to differentiate the sham and IRI even after the injured kidneys were recovered. Altogether, these data indicate that exo-miRs released in renal IRI are associated with TGF-β signaling. Temporal release of exo-miRs which share targets might be a regulatory mechanism to control the progression of AKI.

## Introduction

Acute kidney injury (AKI) is a condition that results in an abrupt decrease in renal function and is now known to be a risk factor for progression of chronic kidney disease (CKD)^1^. Incomplete recovery following AKI is often associated with tubulointerstitial fibrosis and chronic renal inflammation, which likely lead to CKD^2–4^. Currently, we lack specific treatments for AKI or diagnostics for the progression of AKI. Thus, both early detection and progress staging of AKI are an important step in controlling AKI effectively as well as improving clinical outcomes.

Urinary exosomes are extracellular vesicles that originate from the endocytic pathway and are released by fusion of multivesicular endosomes with the apical membrane of renal epithelial cells^5,6^. Urinary exosomes are constitutively released from epithelia, and the repertoire of their cargoes is likely selectively determined in response to the specific causes of kidney injury, thereby allowing urinary exosomes to potentially serve as biomarkers. Indeed, the intracellular traffic of cargo proteins depends not only on the endocytic pathway but also on the biosynthetic-secretory pathway for their exosome release^7^, suggesting that the cargoes in exosomes can mirror the cellular states of exosome producing cells.

In addition to the protein cargoes, almost all classes of RNAs, including miRNA (exo-miRs), are loaded in exosomes. In exosomes are present pre-miRNAs, as well as mature miRNAs, which undergoes exosome-associated miRNA processing^8^. These miRNAs are packaged inside exosomes or appear in isolated exosomes via the association with exosome surface^9^. miRNAs are endogenous small regulatory RNAs that post- transcriptionally/translationally downregulate protein expression by binding of their seed sequence to the target mRNA^10^. Excitingly, intercellular transfer of exo-miRs has been reported to elicit gene expression changes in the recipient cells^11,12^. However, the intercellular transfer role of exo-miRs under physiological conditions still needs to be thoroughly investigated, considering the relative copy number of miRNAs transferred to target mRNAs in the recipient cells for their gene expression regulation.

## Materials and Methods

### NRK52E and mIMCD3 cell culture

NRK52E cells (CRL-1571) were purchased from ATCC and were cultured in Dulbecco’s modified Eagle’s medium, DMEM (30-2002, ATCC), and supplemented with 5% fetal bovine serum (Gemini), 50 units/ml penicillin (Gibco), and 50 ug/ml streptomycin (Gibco). mIMCD-3 cells were obtained from UCSF cell culture core and were cultured in DMEM:F12 (30-2006, ATCC), supplemented with 5% fetal bovine serum (Gemini), 50 units/ml penicillin (Gibco), and 50 ug/ml streptomycin (Gibco).

### Animals and bilateral renal ischemia/reperfusion injury

All animal studies were conducted with approval from the University of Miyazaki in accordance with the University Guidelines for Institutional Care and Use of Laboratory Animals. Male rats (Sprague-Dawley, SD, 10 wks) were purchased from Kyudo (Saga, Japan) and the rats were randomly divided into two groups: a sham operation group and a renal ischemia/reperfusion injury (IRI) group subjected to an IR operation. In the operation for bilateral renal IR, the left and right renal vascular pedicles were clamped using two microvascular clamps (Roboz, Gaithersburg, MD) for 25 minutes, and then the kidneys were reperfused with blood. The sham operation was performed in the same way without clamping of the renal pedicles. The day of the operation for IR was designated as day 0. All the animals used in this study had free access to clean water and a normal diet. Blood samples were collected from the lateral tail vein. The concentrations of plasma urea nitrogen and creatinine were measured using autoanalyzer (DRY-CHEM 3500i, Fuji Film Medical, Tokyo, Japan) and urine osmolality was measured using an automatic osmometer (OM-6060, Arkray, Inc., Kyoto, Japan).

### RNA extraction from exosomes, kidneys, and cell lines

**Figure 1:**
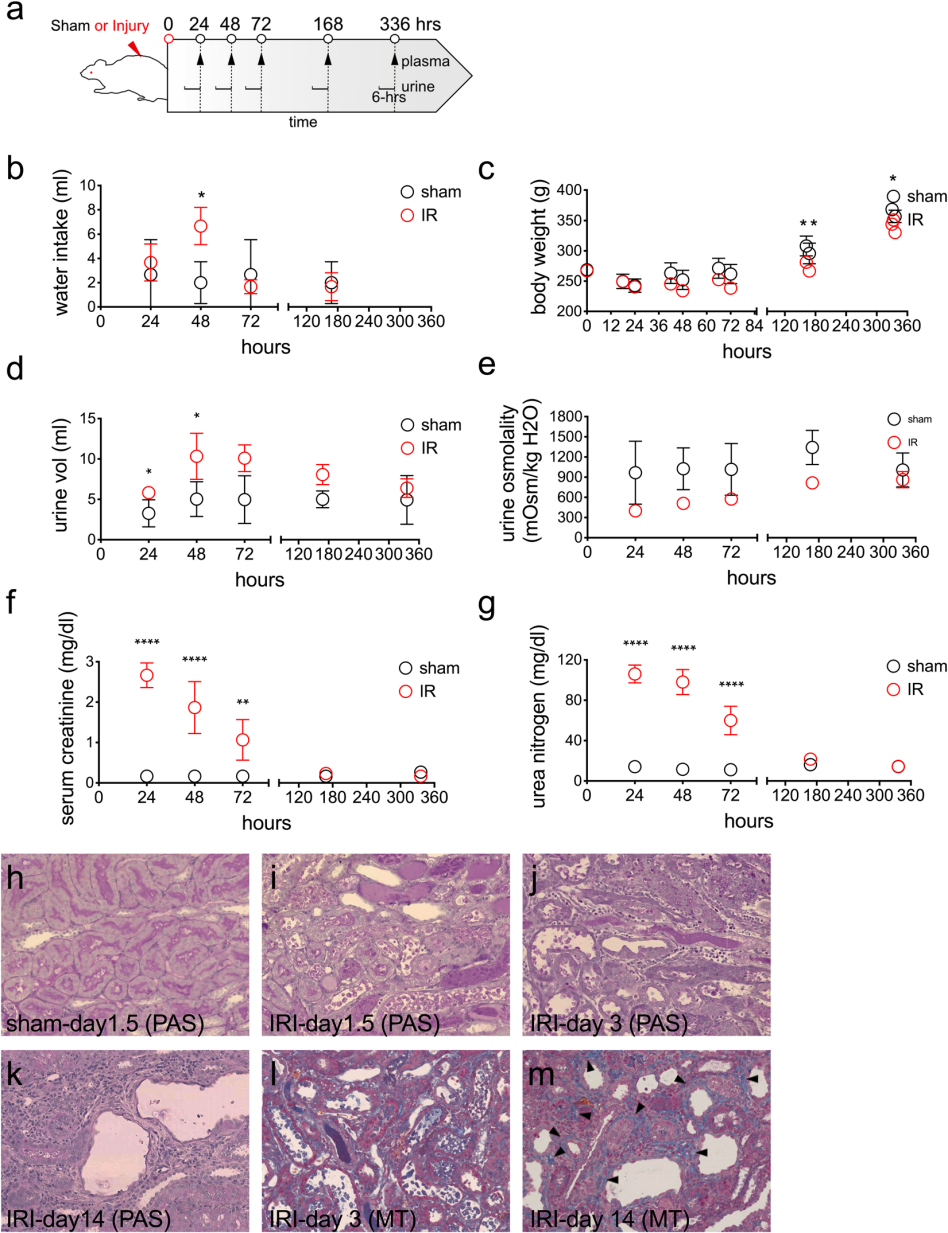
Rat kidney injury following bilateral renal ischemia/reperfusion. Experimental design (**a**) and the longitudinal changes of water intake (**b**), body weight (**c**), urine volume (**d**), urine osmolality (**e**), serum creatinine (**f**), and urea nitrogen (**g**) in sham and bilateral renal ischemia/reperfusion injury (IRI). Data are means ± SD. n = 3 for each group (sham and IR). Significance was calculated using 2-way ANOVA and labeled as *P < 0.05 **P < 0.01 ***P < 0.001 in graphs. Representative histological images of the renal outer medulla are shown in h-m. Kidney sections from rats at 1.5 days (**h**) after sham surgery or 1.5 (**i**), 3 (**j**), and 14 days (**k**) after IRI surgery were stained with periodic acid-Schiff (PAS). Also, kidney sections from rats 3 (**j**), and 14 days (**k**) after IRI surgery were stained with Masson’s trichrome (MT). Arrow heads indicate the interstitial fibrosis region. Bars = 200 μm.

6-hour urine collection was done at the time points shown in Fig. 1a, and collected urine was immediately stored at −80 °C until RNA extraction. Frozen urine samples, each with a volume of 1 ml, were thawed on rotator for 15 min and then centrifuged at 17,000 x g for 10 min. Thereafter, small RNAs in urine were extracted, using the Urine exosome purification and RNA isolation kit (Norgen Biotek) according to the manufacturer’s instructions. For urinary exosome analysis for RT-PCR, urine samples were subjected to the total exosome isolation reagent from urine (Thermo Fisher) and then RNAs were extracted using miRNeasy kit (Qiagen). For exosome-loaded RNAs from conditioned medium, collected conditioned medium was centrifuged at 2000 × g for 15 min and then 10,000 × g for 20 min. The resulting supernatant was combined with 0.5 volume of Total Exosome Isolation reagent (Invitrogen), mixed by vortexing. After incubation at 4 °C for 24 hrs, exosomes were pelleted by centrifugation for 1.5 hrs at 10,000 × g. miRNAs were isolated as described above. For microarrays of kidney miRNAs, total RNA was isolated from whole kidney using TRIzol reagent (Invitrogen) and miRNAs are then purified using miRNeasy mini kit (Qiagen).

### RNA-sequencing and data analysis

After building small RNA libraries using QIASeq miRNA library kit (Qiagen), small RNAs were sequenced and miRNAs were recognized using CAP-miRseq pipeline^13^. Briefly, adaptor and primer sequences from the library were trimmed using Cutadapt, sequence reads were then aligned to the rat genome (version 5) using Bowtie and miRDeep2 mapper, gene-level count data were generated using HTSeq, and finally differential expression analysis were performed using EdgeR^14^. For the over-represented motif search, 75 miRNAs found in urinary exosomes whose total sequence count is more than 3000 and 11 miRNAs not found in urinary exosomes were collected. Using these two datasets, we ran the OOPS model of Multiple Em for motifi Elicitation with discriminative mode to find 4–6 nucleotide long motifs. The identified motif had the lowest E-value in this search setting. The RNA-seq dataset is provided in Supplementary table 1 and was deposited in GEO (GSE124669).

### Microarray analysis of miRNAs in(side) kidneys

RNAs isolated from each sample were labeled using FlashTagTM Biotin HSR Labeling kit and hybridized to an Affymetrix GeneChip® miRNA 4.0 Array according to the manufacturer’s instructions. miRNA expression in kidneys was analyzed using Affymetrix Transcriptome Analysis Console 4.0. The microarray dataset is provided in Supplementary table 1 and was deposited in GEO (GSE125305).  

### Principal component analysis

Principal components were calculated using pcaMethods R package^15^.

### Heatmap

Differentially expressed miRNAs between sham and IRI groups from each time points were plotted in a heatmap, and Euclidean distance metric was used for hierarchical clustering, using MEV^16^.

### Reverse transcription- quantitative polymerase chain reaction (RT-qPCR)

12.5 ng of RNAs isolated from urinary exosomes were used to generate poly(T) primed cDNA and then miRNA expression analyses were performed according to miRCURY Locked Nucleic Acid (LNA) miRNA PCR assays (Qiagen) or custom primers for U6 RNA Forward : 5′-gcttcggcagcacatatacta-3′; Reverse, 5′-cgaatttgcgtgtcatccttg-3′). SYBR Green- based real-time quantitative PCR (qPCR) detection (NEB) was performed using CFX96 touch PCR machine (Bio-Rad). To determine TGF-β1- regulated miRNA expression in NRK52E and mIMCD3 cells, 1.0 × 10^6^ cells were starved for 24 hours in serum-free medium and were then treated with 10 ng/ml of TGF-β1 (R&D System) for the time points described in Fig. 5. 15 ng of total RNAs were used to generate poly(T) primed cDNA and then miRNA expression analyses were performed according to miRCURY LNA miRNA PCR assays (Qiagen). qPCR detection was performed using CFX96 touch PCR machine.

### Western blot analysis

For total protein preparation, cells were lysed in 65.8 mM Tris-HCl (pH 6.8), 26.3% glycerol, 2% SDS, 50 mM 2-mercaptoethanol, and Complete protease inhibitor cocktail (Roche). For exosome marker analysis, proteins in total cell lysates, microvesicle fraction (10,000 x g pellet during the exosome isolation described above), or exosome fractions were separated by 4–12% SDS-PAGE and subsequently transferred electrophoretically to nitrocellulose membrane. The blots were probed with anti-syntenin (Bio-Rad), anti-Alix (Cell Signaling Technology), or anti-Rictor (Cell Signaling Technology) as primary antibodies followed by appropriate HRP-conjugated secondary antibodies for chemiluminescence detection. Unsaturated signals on the blots were captured with Odyssey Fc Imaging System (Li-Cor).

### Histology

Histological sections were stained with periodic acid-Schiff (PAS) or Masson’s trichrome (MT) reagents (Muto Pure Chemicals Co., Ltd., Tokyo, Japan), and microscopic observation of the stained specimen was performed as previously described^4^.

## Results and Discussion

To test our hypothesis that exo-miRs released during renal injury could mirror the cellular states of the kidneys, bilateral renal IR surgery was performed in rats, and plasma and urine samples were collected longitudinally at the indicated times following the surgery (Fig. 1a). As shown in Fig. 1f,g, plasma creatinine and urea nitrogen concentrations in the IR group were significantly increased at 1, 2, and 3 days after the surgery and thereafter were restored to the normal levels, when compared with those in the sham group. Overall, the measurements of water intake, urine volume, body weight, and urine osmolality were not dissimilar between the sham and the IR groups except that water intake at 2 days, urine volume at 1 and 2 days,  and body weight at 7 and 14 days after the surgery were significantly different between the sham and the IR group.

Histological examination revealed that IRI caused tubular cell necrosis, loss of brush border, cast formation at 1.5 days after the surgery (Fig. 1i). At 3 days after the surgery, dilation of tubular lumen and cell infiltration in the interstitial region were also observed (Fig. 1j). At 14 days after the surgery, morphological changes such as tubular cell necrosis and cast formation became less, and dilation of tubular lumen, large nuclei in tubular cells, and renal interstitial fibrosis were increased (Fig. 1k,m). These results indicate that in our bilateral renal IR model, acute kidney injury occurred in the early phase, accompanied by an initial urinary concentration defect, and renal regeneration and fibrosis developed following the injury (Fig. 1b–m).

**Figure 2:**
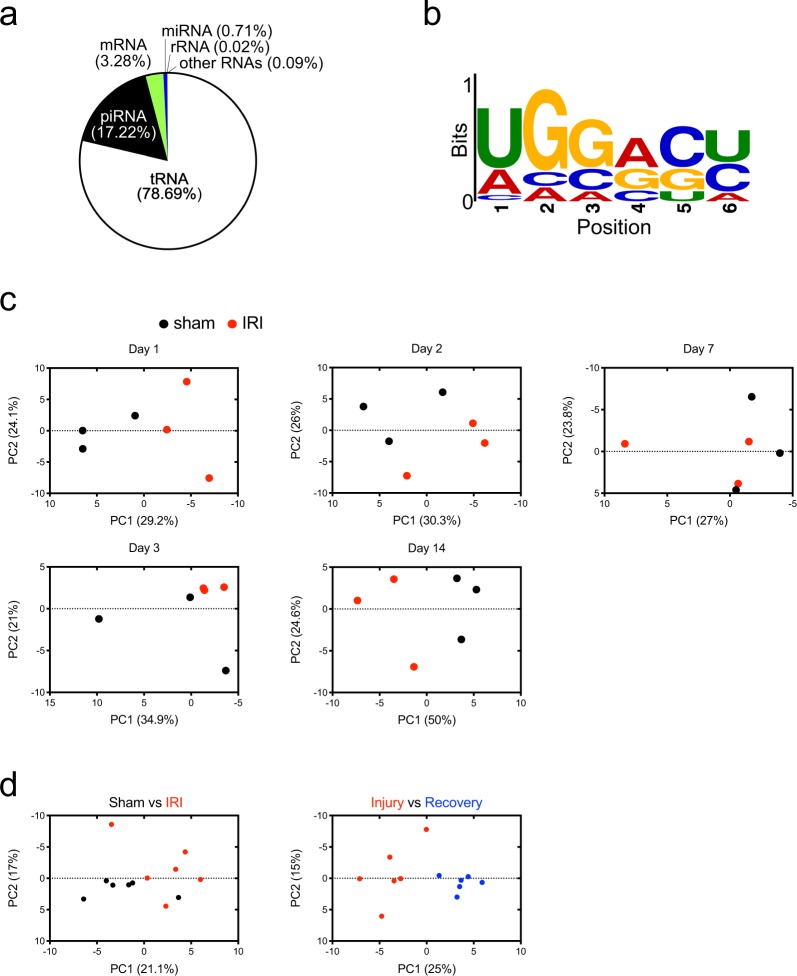
Global similarity and dissimilarity in urinary miRNA transcriptome during the time course of renal IRI. (**a**) Pie chart representation of the distribution of RNA species mapped to known RNAs found in rat urinary exosomes. (**b**) Over-represented sequences in exosome-loaded miRNAs (exo-miRs). (**c**) Principal component analysis (PCA) plots of exo-miR transcriptomes at the indicated time points following sham or renal bilateral IR. n = 3 for each group. (**d**) PCA plot representing exo-miR transcriptomes from the sham and IRI groups at the injury state (day 1 and day 2) (left) and PCA plot representing exo-miR transcriptomes from the injury (day 1-to-2 post IR) and the recovery (day 7-to-14 post IR) in the IR group (right). n = 6 for each group.

To determine miRNAs in the urinary exosomes during IRI, small RNAs in the urinary exosomes isolated at the time points shown in Fig. 1a were sequenced. Western analysis with exosome markers, including Alix and TSG101 was used to confirm that the isolated samples indeed contained exosomes (not shown). The distribution of RNA species in urinary exosomes is shown in a pie chart (Fig. 2a). In this experimental setting, we found abundant piRNAs in urinary exosomes, but the precise annotation remains determined, as the majority of annotated piRNAs expressed in somatic tissues can originate from other non-coding RNA fragments^17^. A previous study from immune T cells showed that miRNAs contain the sequence information that controls their sorting into exosomes, which is called EXOmotif, GGAG^18^. To determine if exo-miRs in urine also contain such motifs, we performed an unbiased search for enriched sequences in the miRNAs^19^. For this analysis, we used two sets of miRNA sequences: one from exo-miRs with more than total 3000 read counts over all time points, and the other from miRNAs that did not appear in our sequencing results as control. Interestingly, the enriched sequence of exo-miRs was GGRS (R is A or G, S is C or G), which overlapped with EXOmotif (Fig. 2b), indicating that the enriched sequences are likely used as a “zip code” for exosome targeting in kidney cells as well. Whether this sequence is sufficient to target miRNAs in urinary exosomes remains to be determined.

In order to detect and visualize the overall effect of covariates associated with each experimental group, we performed principal component analysis (PCA) in which variation detected in two dimensions showed segregation in global miRNA transcription of sham and IRI at day 1, day 2, day 3 following the surgery, though there were batch effects within experimental groups (Fig. 2c). Interestingly, the sham and IRI at day 14 were still separated in the first two dimensions while both serum creatinine (sCr) and urea nitrogen levels of the injury group at day 14 were similar to those from the sham group, suggesting that there is still incomplete recovery or ongoing injury at day 14 which is detected at the expression levels of exo-miRs.

Because the sCr levels peaked at day 2 and its levels at day 7 showed a significant decrease to the levels, which became similar in both sham and IRI groups, we considered days 1 and 2 as injury state whereas days 7 and 14 as recovery state. When comparing the global transcription from sham and IRI, we observed that these two groups were separated (Fig. 2d, left). In addition, the PCA can cluster samples into two groups of exo-miRs in the injury state and recovery state (Fig. 2d, right). Altogether, these data suggest that exo-miRNAs can differentiate between the injury and recovery states, thereby potentially serving as a biomarker source for the progression of kidney injury.

**Figure 3:**
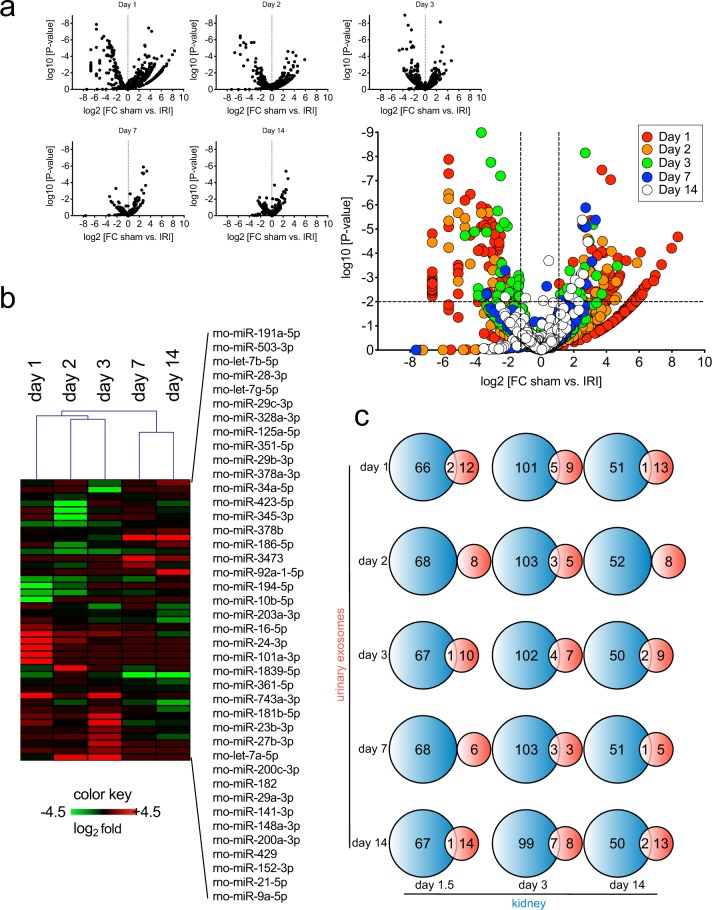
Injury state-specific and recovery state-specific miRNAs in urinary exosomes after renal IR . (**a**) Volcano plots of urinary exo-miR expression at the indicated time points following renal IR and combined all time points. The significance values and fold changes of all expressed miRNAs were computed using EdgeR. (**b**) Heat map with hierarchical clustering of differentially expressed genes with FDR < 0.01 in at least one time point. (**c**) Venn diagram representing overlap of differentially expressed miRNAs in kidney at 1.5 days, 3 days, 14 days post renal IR with differentially expressed urinary exo-miR at 1 day, 2 days, 3 days, 7 days, and 14 days post renal IR.

We determined differentially expressed exo-miRs between the sham and IRI groups over time, and the whole distribution of differentially expressed exo-miRs was visualized in the volcano plots (Fig. 3a). We then clustered samples, based on exo-miR expression changes over time. In this analysis, we applied a false discovery rate of 0.01 for the selection of differentially expressed exo-miRs. All differentially expressed exo-miRs over time with the criteria were plotted with a heatmap, together with hierarchical tree of sample clusters shown in Fig. 3b. Differential gene expression between day 2 and day 3 or between day 7 and day 14 had the least dissimilarity while sample clusters between day 1-to-3 and day 7-to-14 had the most dissimilarity among the time points, which is consistent with the injury and functional recovery states, based on the observed sCr and BUN levels. In other words, the observed expression patterns of exo-miRs over time can cluster into the injury and recovery status following IR.

Next, in order to correlate the expression changes of exo-miRs in urine and kidneys, we compared the exo-miR dataset at the indicated time points with the miRNAs differentially expressed in(side) kidneys at day 1.5, day 3, and day 14 post IRI. The miRNAs in the IRI group were selected by using a fold change threshold of at least 2 and a nominal *P* value cutoff of 0.05 in comparison with the sham group (n = 3 for each group at each time point). Qualitative comparisons were made using Venn diagrams to graphically show the number of miRNAs that are common and those that differ between urinary exosomes and kidney cells (Fig. 3c). While several cellular miRNAs modulated day 3 post IRI appeared in exo-miRs collected over the indicated time points, most of the differentially expressed kidney miRNAs were not overlapped with exo-miRs, suggesting that release of most exo-miRs is determined not only by expression changes but also by regulated intracellular traffic or sorting. Alternatively, the limited overlap of differentially expressed miRNAs in kidney and urine might come from their restricted expression in certain nephron segments.

**Figure 4:**
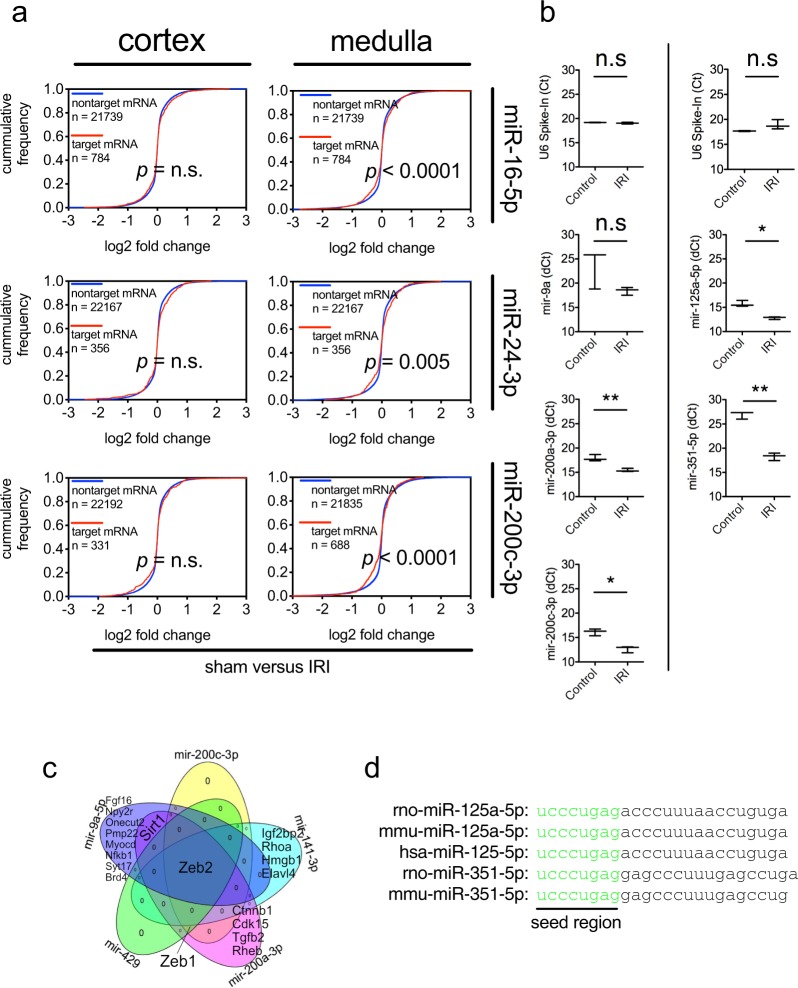
Renal IRI-specific miRNAs with their target mRNAs. (**a**) Comparison of the cumulative distribution of non-target and target mRNAs against the indicated urinary miRNAs in mRNA profiling data of rat kidneys from a day after sham or bilateral IR. Significance was calculated using Kolmogorov-Smirnov test and the values were shown in each graph, if P < 0.05. (**b**) miRNAs upregulated at day 3 and their fold changes in the renal IRI group referenced to sham group. miRNA levels in urinary exosomes were quantified using RT-qPCR. Spike-in raw Ct values serve as control and miRNA levels are expressed as dCt. (**c**) Venn diagram representing overlap of validated targets of mir-9a-5p, mir-141-3p, mir-200a-3p, mir-200c-3p, and mir-429. (**d**) Conserved seed sequences of miR-125a-5p and miR-351-5p, both of which were increased in the recovery state following renal IR.

To determine if exo-miRs (miR-16-5p, miR-24-3p, and miR-200c-3p) which were increased at the early injury state (day 1 after IRI) exerted expression changes of target mRNAs in the kidneys, we searched target mRNAs against these all miRNAs from 14 different miRNA-target interaction databases through the R package MultiMir^20^ with a stringent search condition (e.g. only with top 10% predicted binding strength of each database search). Cellular mRNA transcriptomes of the cortical and medullary rat kidney at day 1 post renal bilateral IRI were collected from the Gene Expression Omnibus (GEO) database (GSE27274). When comparing with cellular mRNA profiling of the cortical and medullary rat kidney, we found 15.3% (247/1614) and 4.1% (66/1615) of the mRNAs targeted by these exo-miRs were down-regulated in the cortical, and medullary kidneys, respectively. Next, the cumulative abundance changes of target mRNAs for each individual exo-miR were compared with those of non-target ones, in order to determine statistically if each exo-miRs affected gene expression of target mRNAs. For this analysis, predicted target mRNAs for each exo-miRs were collected from in silico search using TargetScan^21^. Using the expression mRNA datasets, the cumulative distribution of non-target and target mRNAs from rat kidneys were plotted in Fig. 4a. Notably, the comparison of the effects of IRI relative to sham showed that increased miR-16-5p, miR-24-3p, miR-200c-3p in urine were significantly associated with reduced target mRNAs in the medulla. In contrast, the changes in distribution of the target mRNA in cortex was not statistically significant. These data suggest that miRNA profiling of urinary exosomes could mirror the cellular gene expression in kidneys, which additionally supports a proof of concept that exo-miRs can be considered as a biomarker resource for AKI.

Hierarchical clustering identified two groups of exo-miRs differentially expressed in recovery state with similar expression patterns over time. First, all miR-200 family (miR-141-3p, miR-200a-3p, miR-200c-3p, miR-429), miR-148a-3p, and miR-9a-5p were maximally increased together in urine at day 3. To test whether the differentially expressed miRNAs are loaded in exosomes as well as confirm the results from RNA-seq, we isolated exosomes with polymer-based precipitation as an alternative method and measured expression changes of the miRNAs. RT-qPCR analysis with select exo-miRs from the alternative isolation method confirmed the expression changes observed from RNA-seq (Fig. 4b). Because these exo-miRs were increased at the same time, we searched if these exo-miRs regulated the common targets. Except mir-148a-3p, we were able to retrieve experimentally validated mRNA targets using miRTarBase^22^. For collected experimentally validated targets, see references in^23–34^ for targets of miR-9a-5p^35–39^; for miR-141-3p^38,40–46^; for miR-200a-3p^38,44,47^; for miR-200c-3p^38^; for miR-429. Overlapped targets were represented graphically as a Venn diagram. Indeed, Zeb1/2 were common targets for these exo-miRs (Fig. 4c). These mRNA targets are well- characterized regulators of TGF-β1 signaling^48,49^. Furthermore, the miR-200 family has been reported to regulate renal tubular epithelial-to-mesenchymal transition through TGF-β1 signaling in fibrotic kidneys as well as cell culture^38,50^. Next, miR-125a-5p and miR-351-5p increased simultaneously, at the recovery state, which have the conserved same seed sequence (Fig. 4d). The disease progression of AKI also can depend on the severity of IRI, including ischemic injury time, and AKI can be induced in different ways. It is also possible the role of these exo-miRs may be different, depending on the cause and severity. It will be interesting to know whether these differentially expressed exo-miRs can mirror the injury and the recovery status in other AKI.

**Figure 5:**
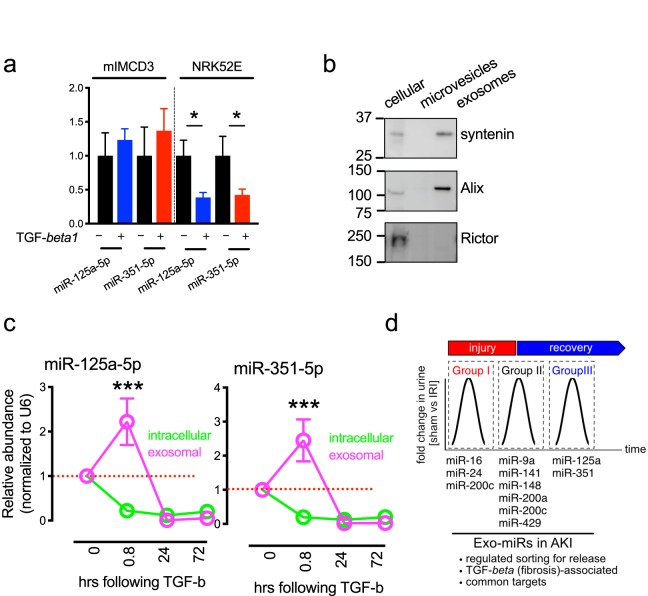
TGF-β1-regulated exosomal release of miR-125a-5p and miR-351-5p. (**a**) RT-qPCR analysis of miR-125a-5p and miR-351-5p expression in NRK52E and mIMCD3 cells treated with 10 ng/ml of TGF-β1 for 48 hours. miRNA levels were normalized using U6 RNA as endogenous control. Data are means ± SD. n = 3 for each group. Significance was calculated using two-tailed t-test and labeled as *P < 0.05 in graphs. (**b**) Western analysis showing indicated proteins in total lysates, microvesicles, and exosome fractions from NRK52E cells. Marker analysis showed enriched exosome proteins in the exosome fraction. Rictor was used as negative control. (**c**) RT-qPCR analysis of miR-125a-5p and miR-351-5p expression in NRK52E cells treated with 10 ng/ml of TGF-β1 and exosomes isolated from the conditioned media at the indicated times. miRNA levels were normalized using U6 RNA as endogenous control. Significance was calculated using 2-way ANOVA and labeled as ***P < 0.001 in graphs. (**d**) Schematic representation of exo-miR release during acute kidney injury. Differentially expressed exo-miRs during injury or recovery state following AKI were listed. Urinary Exo-miRs released during recovery state can mirror TGF-β signaling in ischemia-reperfusion induced AKI and TGF-β signaling controls release of exo-miRs in urine.

Because the exo-miRs released during the early recovery state are associated with TGF-β1 signaling, we tested if expression of miR-125a-5p and miR-351-5p are also regulated by TGF-β1. Since the proximal tubule in kidneys is a major site of injury and repair following AKI^51^, NRK52E cells (proximal tubule cell line) were treated with TGF-β1 for 2 days (Fig. 5a). RT-qPCR analysis showed that both miR-125a-5p and miR-351-5p were significantly downregulated in NRK52E cells, but not in mIMCD3 cells which originate from collecting ducts. Interestingly, the expression of miR-125a-5p and miR-351-5p in NRK52E cells was negatively correlated with that in urinary exosomes. To test if exosome release leads to decreased expression of miR-125a-5p and miR-351-5p in the cells, we measured the time course of miRNA expression in cells as well as exosomes from the conditioned media. Immunoblotting analysis showed that exosome marker proteins, including syntenin^52^ and Alix^53^ were enriched in exosomes fraction but not in microvesicle fraction, validating the exosome isolation protocol used in this study (Fig. 5b). RT- qPCR analysis with cellular and exosome fractions showed that both intracellular exo-miRs were depleted at 45 min after TGF-β1 treatment and thereafter remained unchanged while their expressions in exosomes were peaked at 40 min, indicating that increased extracellular release of these miRNAs led to their reduction in cells (Fig. 5c). Thus, this data suggests that exosomal release could be a mechanism that control miRNA availability and abundance in cells.

In this study, we longitudinally measured microRNAs in urinary exosomes released during renal IRI (Fig. 5d). In kidneys, regulated sorting of exo-miRs is likely determine their exosomal release to urine. Injury-specific exo-miRs were associated with target mRNA expression distributions in renal medulla while recovery- specific exo-miRs had common target mRNAs involved in TGF-β signaling, based on sequence complementarity. Lastly, we demonstrated that exosome release of miRNAs might be a mechanism that controls miRNA availability and abundance in cells. Thus, regulated release of exo-miRs in urine reflects TGF-β signaling in kidneys, which might be useful in noninvasively detecting the disease progression of AKI and AKI-to-CKD.

## Supplementary information

Supplementary table 1
